# Preoperative embolization and immediate removal of a giant pituitary adenoma: a case report

**DOI:** 10.1186/s13104-017-2383-5

**Published:** 2017-01-26

**Authors:** Shunsuke Omodaka, Yoshikazu Ogawa, Kenichi Sato, Yasushi Matsumoto, Teiji Tominaga

**Affiliations:** 1 Department of Neurosurgery, Kohnan Hospital, 4-20-1 Nagamachiminami, Taihaku-ku, Sendai, Miyagi 982-8523 Japan; 2 Department of Neuroendovascular Therapy, Kohnan Hospital, Sendai, Japan; 3 Department of Neurosurgery, Tohoku University Graduate School of Medicine, Sendai, Japan

**Keywords:** Giant pituitary adenoma, Meningohypophyseal trunk, N-butyl cyanoacrylate, Preoperative embolization

## Abstract

**Background:**

Giant pituitary adenomas, with maximum diameter of at least 40 mm, continue to involve high surgical risks despite recent advances in microsurgical and/or endoscopic surgery. We treated a case of giant pituitary adenoma with preoperative endovascular embolization in an attempt to reduce blood loss.

**Case presentation:**

A 48-year-old Japanese Woman presented with severe right visual disturbance. Magnetic resonance imaging revealed a giant pituitary adenoma with maximum diameter of 82 mm. Angiography revealed significant tumor stain, with blood supply mainly from the branches of the right meningohypophyseal trunk. These feeding arteries were endovascularly embolized with n-butyl cyanoacrylate. Subsequently, the tumor was safely removed by transsphenoidal surgery in two stages. The patient showed significant improvement of visual disturbance postoperatively, and was discharged without other neurological deficit. The surgical policy was explained preoperatively to the patients and written informed consents were obtained.

**Conclusions:**

Preoperative embolization of a giant pituitary adenoma is a useful procedure that can potentially decrease the morbidity and mortality of this devastating tumor.

## Background

Pituitary adenomas are benign and grow slowly, but can extend and infiltrate into extrasellar areas, and may become very large before diagnosis. Pituitary adenomas with a diameter of more than 40 mm are classified as giant adenomas, and account for about 10% of all surgically treated pituitary adenomas [1–3]. Giant adenomas are associated with a higher frequency of neuro-ophthalmological symptoms, and postoperative pituitary apoplexy from this tumor is usually associated with very poor outcomes due to significant postoperative tumor swelling, hemorrhage, infarction, necrosis precipitating further mass effect, cerebral edema, and herniation syndrome [1, 4, 5]. The choice of the most appropriate surgical strategy should depend on the need to remove as much tumor as possible to improve the clinical symptoms and to minimize the risk of side effects.

Preoperative embolization of extra-axial tumors, such as meningiomas, is effective for decreasing blood loss, softening the tumor, and facilitating the surgery [6]. However, preoperative endovascular embolization has not been reported in giant pituitary adenomas. We describe a case of giant pituitary adenoma treated with preoperative endovascular embolization, followed by successful removal via the transsphenoidal route, followed by neurologic improvement of the patient.

## Case presentation

### History and examination

**Fig. 1:**
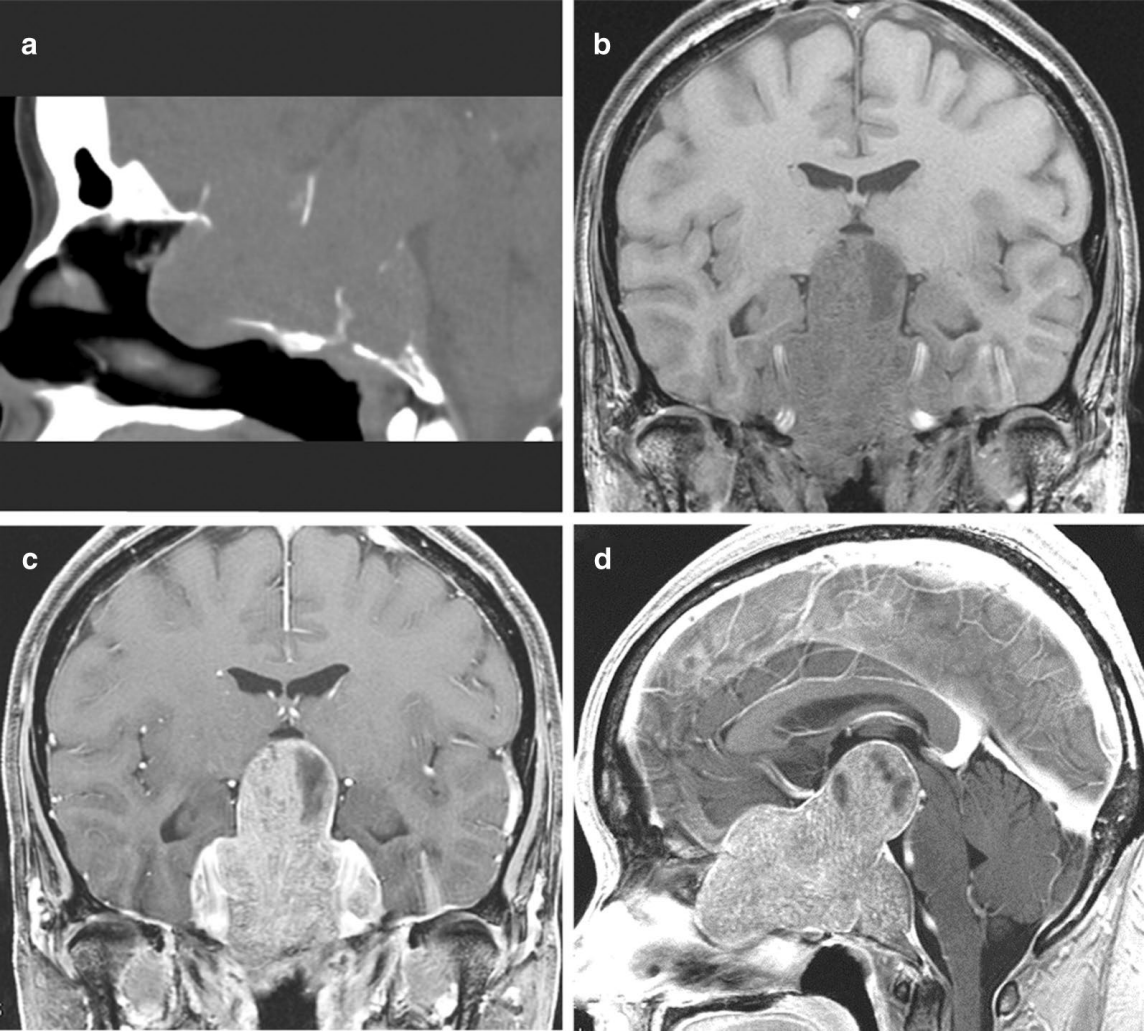
**a** Sagittal bone computed tomography image showing extensive destruction of the sella turcica. **b** Coronal T1-weighted magnetic resonance image showing hypointense giant pituitary tumor. **c, d** Coronal (**c**) and sagittal (**d**) contrast-enhanced T1-weighted magnetic resonance images showing marked enhancement of the tumor

A 48-year-old Japanese Woman presented with a 4-month history of progressive right visual disturbance. She had no history of other diseases. Ophthalmic examination revealed decreased visual acuity in both eyes. The right visual field showed generalized depression, and the left visual field showed temporal hemianopsia. Neurological examination found no other abnormalities. Computed tomography revealed a huge sellar tumor with extensive destruction of the midline skull base (Fig. 1a). Magnetic resonance imaging showed a well-demarcated mass with maximum diameter of 82 mm, extending from the sella to the interpeduncular cistern, with extensive destruction of the clivus. The both intracavernous carotid arteries were totally encased, and the roofs of the both cavernous sinuses were elevated superiorly. The tumor appeared hypointense on T1-weighted images and isointense on T2-weighted images, with marked enhancement with gadolinium (Fig. 1b–d). These neuroimaging features were consistent with giant pituitary adenoma.

**Fig. 2:**
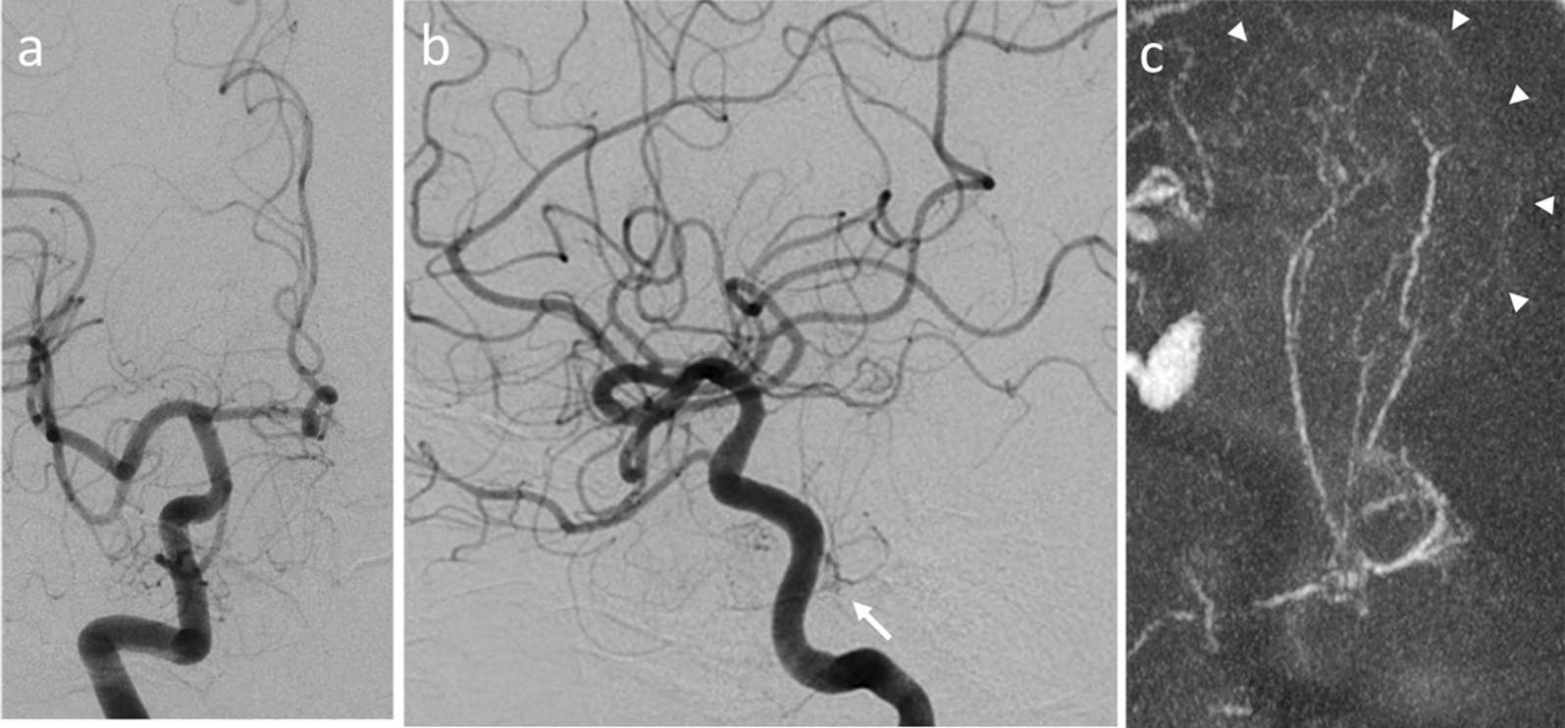
**a, b** Right internal carotid angiograms, anteroposterior (**a**) and lateral (**b**) views, showing the tumor mainly fed by the right meningohypophyseal trunk (*arrow*). **c** Sagittal maximum-intensity projection cone-beam computed tomography image showing a giant hypervascular pituitary tumor (*arrowheads*) with large feeders from the right meningohypophyseal trunk

All biochemical parameters and hormonal status were within the normal ranges except for slight elevation of the prolactin value. Cerebral angiography demonstrated a hypervascular tumor with thick feeding arteries arising from the right meningohypophyseal trunk (MHT) (Fig. 2). The tumor was also fed partially by the right inferolateral trunk, vidian artery, middle meningeal artery, left MHT, and inferolateral trunk. Preoperative embolization was planned based on the presumed diagnosis of giant pituitary adenoma with hypervascular nature.

### Preoperative embolization

**Fig. 3:**
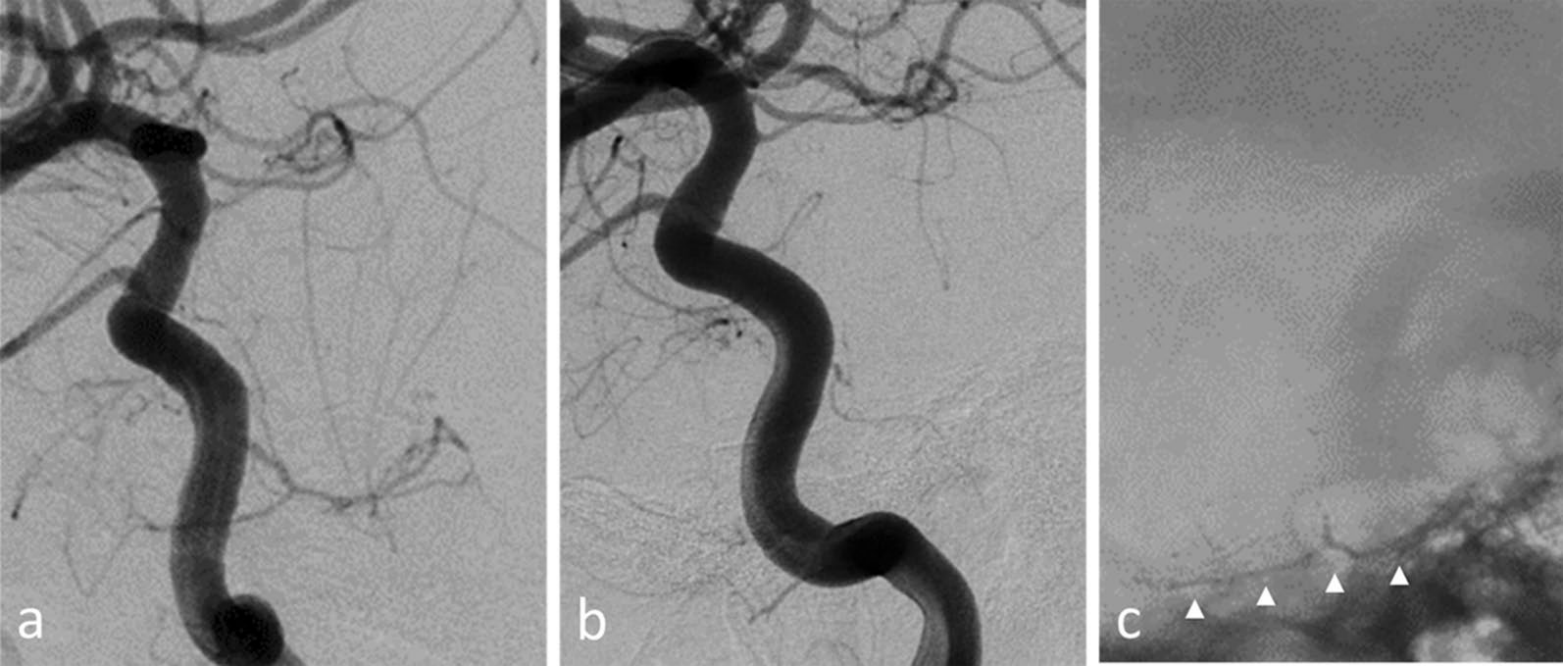
**a, b** Right internal carotid angiogram, oblique view, before (**a**) and after (**b**) embolization of the right meningohypophyseal trunk, showing the marked devascularization of the tumor. **c** Skull radiograph, oblique view, after embolization showing the cast of n-butyl cyanoacrylate (*arrowheads*)

Transarterial embolization was performed via the femoral approach under general anesthesia. After general heparinization, a 7-F guiding catheter was inserted into the right internal carotid artery. A Marathon microcatheter (ev3, Irvine, MN, USA) was navigated selectively over a 0.010-inch Chikai microwire (Asahi Intec, Nagoya, Aichi, Japan) into the right MHT branches supplying the tumor. Super-selective angiography demonstrated diffuse opacification of the lesion. These branches were occluded using 15 and 20% n-butyl cyanoacrylate. Post-embolization angiography demonstrated marked devascularization of the tumor (Fig. 3). Additionally, the feeder from the right middle meningeal artery was occluded using microcoils.

### Surgical removal and postoperative course

**Fig. 4:**
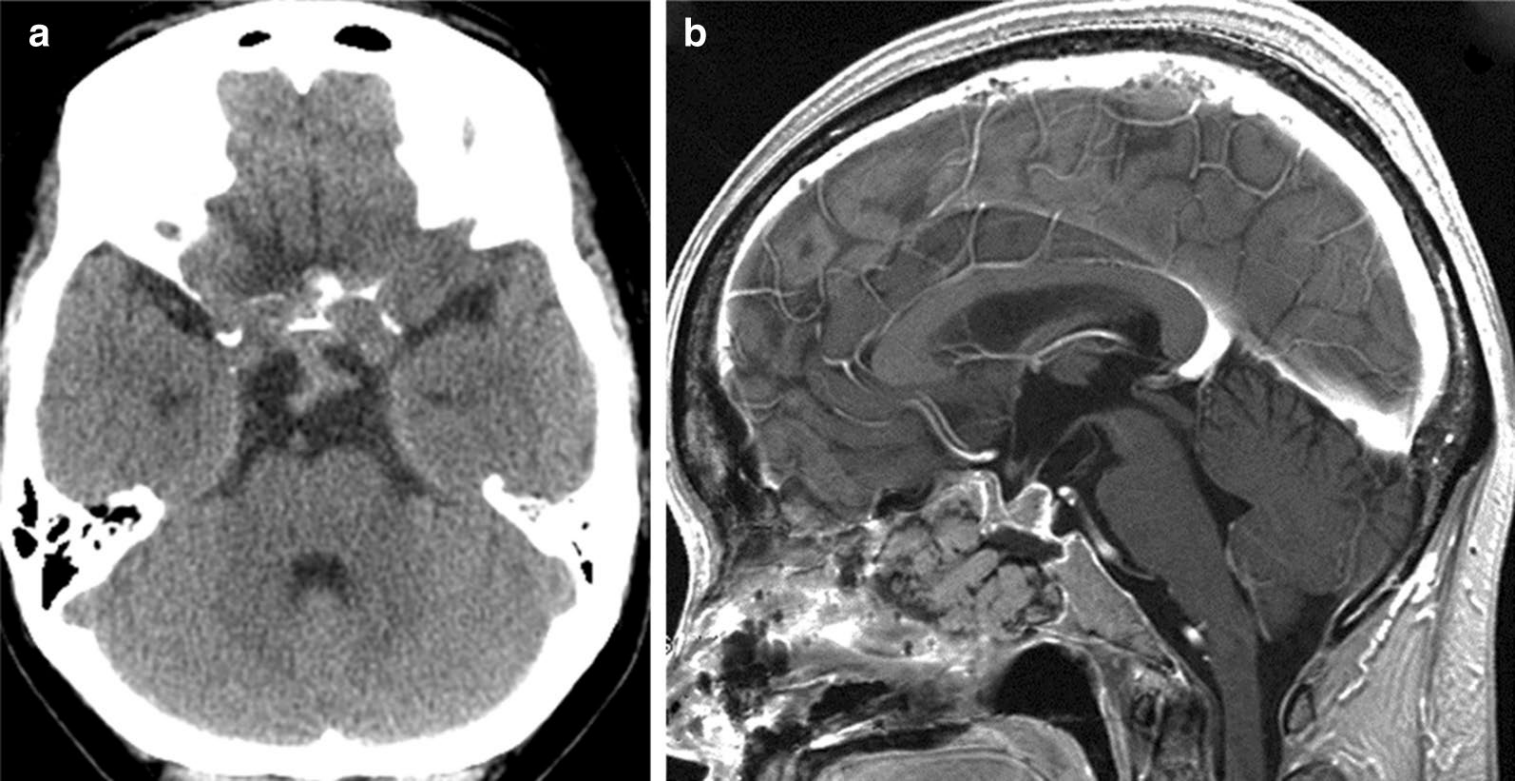
**a** Axial computed tomography image obtained immediately after the first transsphenoidal surgery showing no hemorrhagic complication. **b**: Sagittal contrast-enhanced T1-weighted magnetic resonance image obtained 3 days after the second transsphenoidal surgery showing subtotal removal of the tumor

The tumor removal was performed via an extended transsphenoidal route in a two-stage operation. First surgery was performed on the same day of embolization as gross decompression to avoid mass effect of the tumor due to swelling of necrotizing tissue, and the following surgery was performed 18 days later expected easier removal of the tumor by simple aspiration. And subtotal removal was achieved without complications (Fig. 4). Intraoperative findings showed thick artery (about 500 μm of diameter) and mesh-like thinner arteries (around 150 μm of diameter) ran inside the tumor, which were coagulated and devascularized one by one. The blood loss during the two operations was 671 mL in total. The postoperative course was uneventful, with remarkable improvements in her visual acuity and visual field. The prolactin level was normalized and she had no diabetes insipidus after operation. She was discharged 12 days after the second transsphenoidal surgery. Pathological analysis confirmed the diagnosis of null cell adenoma. The surgical policy was explained preoperatively to the patients and written informed consents were obtained. Overall study design was approved by the Ethical Committee of Kohnan Hospital 2015.

## Discussion with conclusion

The primary goal of surgical treatment of giant pituitary adenoma is improvement of the visual, endocrinological, and neurological symptoms, whilst ensuring the lowest risk for morbidity and mortality rates. However, radical removal of giant pituitary adenomas remains a challenge for neurosurgeons despite the development of microsurgical techniques. Surgical management of giant adenoma has a higher complication rate than that of non-giant adenoma. The reported mortality and morbidity rates in surgical series of giant pituitary adenomas are 0–3.2 and 4.2–15.5%, respectively [1, 2, 7, 8]. Clival invasion is also associated with a higher rate of operative complications [9].

The most likely explanation for the worse surgical outcome is that resection of giant tumors is frequently incomplete, thus putting patients at increased risk of postoperative pituitary apoplexy occurring within the immediate postoperative period [4, 5]. Postoperative pituitary apoplexy is usually associated with very poor outcomes due to significant postoperative tumor swelling, hemorrhage, infarction, necrosis precipitating further mass effect, cerebral edema, and herniation syndrome [1, 4, 5]. Giant pituitary adenomas have much more vascularity than non-giant pituitary adenomas [10], so that postoperative pituitary apoplexy may correlate with such hypervascularity. In the present case, preoperative neuroimaging, including contrast-enhanced magnetic resonance imaging and angiography, suggested that the tumor was hypervascular. Therefore, we performed preoperative embolization followed by two-stage transsphenoidal surgery. Although we could not completely remove the tumor extending into the cavernous sinus and clivus, the postoperative course was uneventful without hemorrhagic complication after both stages of transsphenoidal surgery. We suggest that preoperative embolization of giant pituitary adenoma has the potential to prevent postoperative pituitary apoplexy.

Embolization facilitates tumor resection by limiting blood loss and softening the tumor, which result in a clear operation field and reduced forces transmitted to adjacent neural structures, making surgical resection safer [6, 11, 12]. The usefulness of preoperative embolization through the internal carotid artery branches is much less understood due to the technical difficulties and higher risks compared with embolization through the external carotid artery branches [11, 13]. The risks include thromboembolic events, post-embolization cranial nerve deficits, intratumoral hemorrhage, post-embolization swelling, and general complications related to angiography [13]. The recent development of the high-resolution road mapping system and concurrent development of softer and smaller microcatheters and microwires have enabled super-selective embolization through the internal carotid artery branches [14]. Major blood supply of giant pituitary adenomas originates from branches of the infraclinoidal portion of the internal carotid artery [15]. The MHT is generally very small and arises at an acute angle from the internal carotid artery, and these anatomical relationships create challenges to direct catheterization, but few clinical trials have investigated preoperative embolization of the MHT in meningiomas [13, 14, 16]. Consequently, the surgical indications must carefully balance the risks against the potential benefit.

The present case describes the technique of preoperative embolization of giant pituitary adenoma. No complications were observed and marked devascularization was achieved. The tumor was hypervascular, and was supplied by feeders such as thick branches of the MHT. We used n-butyl cyanoacrylate at a relatively low concentration, because we could insert the microcatheter into a distal portion of the MHT and could deliver embolic material deep inside the tumor. Use of n-butyl cyanoacrylate is very safe for the embolization of head and neck tumors [17]. Moreover, we planned to perform the embolization and the immediate transsphenoidal surgery in a single session before reconstitution of the collaterals to the occluded tumor area. Early surgical tumor removal can also reduce the risk of repeated general anesthesia and reduce other delayed reactions to tumor embolization such as mass effect of the tumor due to swelling with herniation or obstructive hydrocephalus [12]. Ischemic pituitary adenoma apoplexy often leads to the progression of cranial nerve palsy and decreased level of consciousness [18]. Control of intraoperative bleeding was effective, and intraoperative blood loss was low, resulting in successful staged transsphenoidal surgery without complications. The present case demonstrates that preoperative embolization of giant pituitary adenoma should be regarded as a feasible option to decrease surgical blood loss.

In conclusion the present case illustrates the usefulness and feasibility of preoperative embolization of giant pituitary adenomas. Recent advances in microcatheter, microguidewire, and imaging technology may allow the introduction of new therapeutic strategies and indications for these complex lesions.

## Abbreviation

MHT: meningohypophyseal trunk
